# LC-MS/MS Determination of 21 Non-Steroidal Anti-Inflammatory Drugs Residues in Animal Milk and Muscles

**DOI:** 10.3390/molecules26195892

**Published:** 2021-09-28

**Authors:** Marta Pietruk, Piotr Jedziniak, Małgorzata Olejnik

**Affiliations:** 1 Department of Pharmacology and Toxicology, National Veterinary Research Institute, 57 Partyzantow Avenue, 24-100 Pulawy, Poland; jedzi@piwet.pulawy.pl; 2 Department of Omic Analyses, National Veterinary Research Institute, 57 Partyzantow Avenue, 24-100 Pulawy, Poland; 3 Department of Basic and Preclinical Sciences, Faculty of Biological and Veterinary Sciences, Nicolaus Copernicus University in Torun, 11 Gagarina St., 87-100 Torun, Poland; molejnik@umk.pl

**Keywords:** NSAIDs, LC-MS/MS, residues

## Abstract

The presented procedure combines experience from two LC-MS/MS methods previously developed by our team for NSAIDs determination in meat and milk. The novelty was a modification of sample preparation and combining LC-MS/MS method for milk and muscle. The clean-up procedure was investigated, leading to a change from SPE to dSPE with C18 bulk sorbent. Unlike most of the existing methods, chromatographic separation was achieved on a C8 chromatographic column. This method was developed and validated under European Commission Decision 2002/657/EC. Recovery for milk samples values between 86.3% to 108%, with the coefficient of variation, varied from 5.51% to 16.2%. The recovery for muscle was calculated to be between 85.0% and 109%, and the coefficient of variation was—4.73% to 16.6%. The validation results prove that the method is suitable for confirmatory purposes in milk and muscle. Of 452 samples tested in 2019 and 2020, two have been identified as non-compliant.

## 1. Introduction

Non-steroidal anti-inflammatory drugs (NSAIDs) are commonly used pharmaceuticals in both human and animal medicine. They can reduce pain, inflammation, prevent blood clots, and decrease fever. NSAIDs are a relatively safe group of drugs. However their usage can cause side effects, including peptic ulcer disease, kidney and liver failure, or hypersensitivity (up to 2% of adults) from which the most dangerous is anaphylactic shock [1]. Despite the similar mechanism of action (inhibition of the enzymes responsible for proteinoid synthesis—COX), NSAIDs are a diverse group of drugs, with different half-lives, from 4 h for ibuprofen, diclofenac or acetaminophen, to 20–60 h for oxicams (in human) [2]. In veterinary medicine, they became an essential class of medications for most mammalian animals. There is still an insufficient amount of information about their pharmacokinetics in animals; however, it has been proven that acidic drugs (pKa 4–5), including diclofenac, ibuprofen, ketoprofen and more, seem to accumulate and persist in inflamed tissue, such as the synovial fluid of inflamed joint [3]. Because the administration of NSAIDs for medical and veterinary purposes is so vast, they are also regarded as environmental contaminants [4]. Recent findings show NSAIDs residues can cause deadly threats for many animal species, such as Indian vultures who die due to diclofenac poisoning [5].

The monitoring of residues in food of animal origin is an important task for both human and animal health. Although the risk associated with the intake of drugs with food is low, it still must be assured that the levels of these substances will not exceed the limits introduced in regulatory documents [6]. Despite some NSAIDs being registered as veterinary drugs, only few have defined maximum residue levels (MRL) (Table 1) and are allowed for use in food-producing animals. According to the European Safety Food Authority (EFSA), in 2019, European laboratories involved in the official food control analysed 24,387 samples (muscle, milk), of which 46 (0.19%) were reported as non-compliant [7].

Many methods for the determination of NSAIDs in animal tissue and milk have been developed. Although some single-residue methods are based on HPLC-UV methods [8], LC-MS/MS has become a technique of choice due to European regulations and their constant evaluation. The development of the multi-residue method for NSAIDs is still a challenge because of their chemical variety. Most of them have a chemical structure of weak acids; however, there are some basic compounds (metamizole and its metabolites). That is why previous methods for the determination of NSAIDs residues in milk were using different sample preparation and analytical conditions for acidic and basic NSAIDs [9,10]. Moreover, analysis of muscle tissue requires sufficient clean-up, including SPE and/or filtration, which makes sample preparation time-consuming and labour-intensive [10,11,12,13,14].

Moreover, some compounds such as phenylbutazone and oxyphenbutazone are highly prone to oxidation during analysis. For this reason, some methods include using ascorbic acid as an antioxidant [9,11,13,14,15]. These challenges resulted in only a few methods that combine two or more different matrices [12,13,16].

The goal of this study was to develop a method that would work for both muscle tissue and milk, for all required compounds, sensitive enough to detect a wide concentration range (0.1 µg/kg for diclofenac and 100 µg/kg for metamizole), with a fast and straightforward sample preparation process.

## 2. Results and Discussion

### 2.1. LC-MS/MS Analysis

NSAIDs are a group of polar compounds; thus, the ESI source is the most suitable choice. It was previously established that the negative ionisation is suitable for more acidic NSAIDs (better signal to noise ratio), and positive ionisation showed better results for metamizole metabolites, some coxibs, and NSAIDs (e.g., diclofenac) [18]. Previous assumptions were confirmed during instrument tuning for each compound when both ionisation modes were tested. For diclofenac, positive ionisation was selected due to better reproducibility results. Most of our results are in accordance with previously published data [19,20].

As a mobile phase, a mixture of MeOH:ACN (8:2) and 0.01 M ammonium formate (pH 5) was chosen. It has been proven that ammonium formate helps with ionisation under ESI (-) mode by changing the pH of the mobile phase [11]. Our team’s proportion of MeOH and ACN was previously optimised, resulting in the highest signals [21]. Although most of the publications imply using the C18 column [9,11,12,13,16,19,22,23], under our mobile phase composition, the best results of separation, better pick shape, and relatively short time of analysis were obtained with the C8 column, which was then used for further optimisation.

### 2.2. Extraction and Clean-Up

Sample preparation is always the crucial point of every method. Most of the published methods focus on one specific matrix. Three papers combine similar sample preparation for muscle tissue and milk [12,13,16]. This is, by far, the most significant change that allowed to speed up the analysis. The literature overview shows that the most used extraction method is liquid–liquid extraction. Due to the different sample weights, the volume of organic solvent was optimised for sufficient extraction yield. This step was already optimised in the previous studies [23]. It was proven that the deconjugation step is needed for some analytes (CPF, FLU, IBU) [21].

For analysis of milk, this approach seems unnecessary. The main route of administration for dairy cows is intramammary, and because glucuronidation occurs mainly in the liver, drugs are less likely to create glucuronides. Although marker residue for FLU in milk is 5-OH FLU, it was proven that administration of FLU could cause the presence of flunixin glucuronide in milk, but the concentration of glucuronide form was not significant enough to re-evaluate the marker residue [21]. It was shown that the extraction process performed in acidic conditions results in conjugate being hydrolysed back to the parent substance [24].

As the previous research proves, the clean-up process is one factor that may improve results and reduce matrix effect, although it is still insufficient to prevent some ion suppression [25]. In some methods, solid-phase extraction (SPE) or dispersive SPE is performed, usually by using sorbents such as C18 [9], polymers [12], or QuEChERS [14,15]. There are, however, methods in which further purification is not performed [16,19]. In the presented method, by implementing dispersive SPE, the time of sample preparation has been reduced by half compared to the traditional SPE [10,12]. The amount of the sorbent was optimised by analysing fortified samples of milk and muscle. Four sorbent weightings were checked (0.25 g, 0.50 g, 0.75 g, 1.00 g). As a result, 1.00 g of sorbent was chosen for further analyses, giving the best compromise between recovery and clean-up efficiency. As an additional step, centrifugation at −20 °C helped remove a large part of the co-extracted fatty acids and water phase.

**Figure 1:**
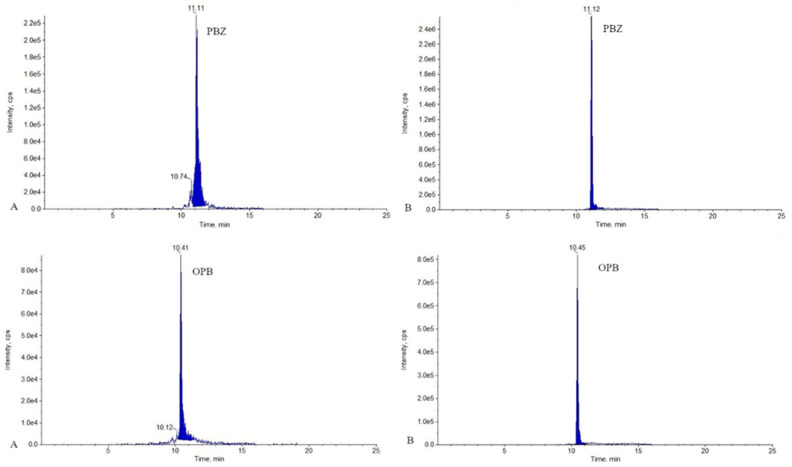
Ion chromatograms for PBZ and OPB (transitions used for quantification) obtained from bovine milk sample spiked at level 1.0 MRL. Sample A was reconstituted in 0.1 mL of mobile phase and sample B in 0.1 mL of DMSO.

Finally, the solvent and solvent volume for samples reconstitution were optimized—DMSO and the mobile phase were verified. Using DMSO slowed down the sample evaporation, which most likely reduces the oxidation of the susceptible compounds. As a result, DMSO showed the best results by improving the signal intensity and peak shape of OPB and PBZ (Figure 1).

### 2.3. Method Validation

Results of the validation experiment for milk and muscle samples were in line with criteria laid down in European Commission documents [26]. During the selectivity experiment using other veterinary drugs, we did not observe any interference peak in the retention time of analytes in the scope of the presented method. The working ranges for both matrices were tested performing the F-test, and results fit the linear regression model. The recovery obtained for milk was in the range of 86.3% for 5-hydroxyflunixin to 108% for both flufenamic (FLUF) and mefenamic (MEF) acids. For precision, the highest values for the coefficient of variance (CV) were obtained for PBZ and DC—17.0% and 16.2%, respectively. For muscle samples, recoveries were all in the range between 85% to 110%, with the lower value of 85.0% for firocoxib to the highest equal to 109% for rofecoxib (ROFE). Given precision (CV%), the highest values were found for 4-MMA—16.6%. Calculated values of CCα and CCβ confirm the method is fit for purpose as a routine method for determining NSAIDs in milk and muscle samples. Results of the validation experiment were in line with work published by other authors [11,16,19]. The most significant discrepancies can be found for precision. Values of coefficient of variance obtained using our method are higher than in other authors’ methods, especially in milk samples (DC, CPF, MEL). It should be noted that PBZ is considered one of the most difficult analytes in the NSAIDs group because it reasonably quickly undergoes oxidation [27]. Diclofenac is also an analytical challenge because its MRL level in milk is significantly lower than other analytes.

### 2.4. Real Samples Analysis

**Figure 2:**
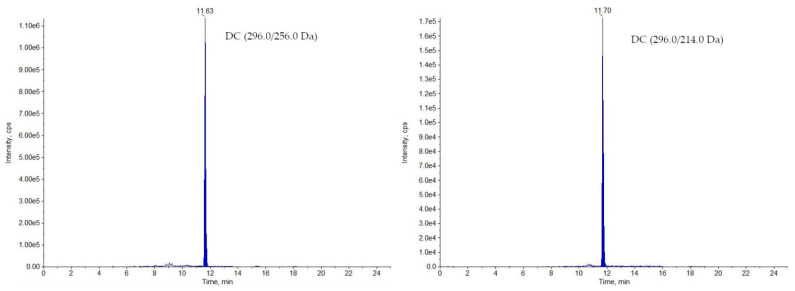
Chromatograms of the non-compliant sample of milk containing DC.

**Figure 3:**
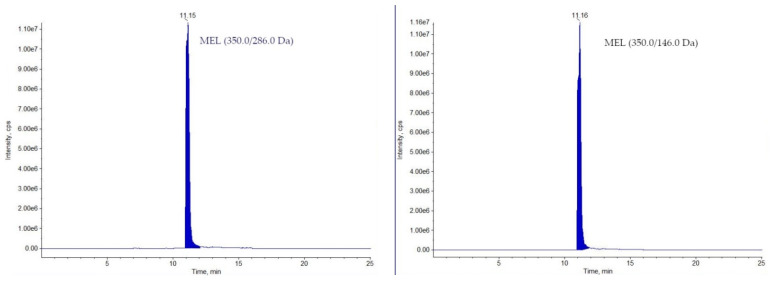
Chromatograms of the non-compliant muscle sample containing MEL.

The developed method has been routinely used for analysing samples collected in National Residue Control Plan in Poland since 2019. In 2019, for 240 analysed samples (including 209 muscle and 31 milk samples), only one milk sample was identified as non-compliant (DC—3.50 µg/kg; Figure 2). The stated concentration was 35 times higher than the MRL value for DC in milk. In 2020, 233 samples were analysed (197 muscles and 36 milk), from which one muscle sample was found non-compliant (MEL—279 µg/kg; Figure 3), exceeding almost 13 times the MRL value for meloxicam in muscle (see Table 1). Although the transgressions were severe, only two samples were identified as non-compliant in a two-years period, which make 0.42% of all analysed samples.

## 3. Material and Methods

### 3.1. Reagents and Chemicals

Acetonitrile (ACN), methanol (MeOH), and dimethyl sulfoxide (DMSO), all three LC-MS grades, were obtained from J.T. Baker, Germany. Formic acid (≥95%), ascorbic acid (puriss p.a.), ammonium formate (puriss p.a.), and β-glucuronidase from *Helix pomatia* (HP-2) were from Sigma-Aldrich, Germany. Merck supplied acetic acid (100%). Ammonium acetate was from Chempur, Poland, sodium acetate (puriss p.a.) was purchased from POCH, Poland. Octadecyl bulk sorbent was from Avantor, USA. Ultrapure water (resistance >18 mΩ) was obtained from the Milli-Q system (Millipore, France). The analytical standards were supplied by the following manufacturers: diclofenac (DC), flunixin meglumine (FLU), 5-hydroxy flunixin (5-OH FLU), carprofen (CPF), ketoprofen (KTP), mefenamic acid (MEF), tolfenamic acid (TOL), niflumic acid (NIF), flufenamic acid (FLUF), meloxicam sodium (MEL), naproxen (NAP), celecoxib (CELE), ibuprofen (IBU), meloxicam-d3 (MEL-d3), diclofenac-13C6 (DC-13C6), phenylbutazone (PBZ), flunixin-d3 (FLU-d3), phenylbutazone-13C12 (PBZ-13C12), 4-aminoantipyrine (4-AA)—Sigma-Aldrich, Germany; rofecoxib (ROFE), oxyphenbutazone monohydrate (OPB), firocoxib (FIRO), 4-methylaminoantipyrine (4-MAA), 4-formylaminoantipyrine (4-FAA), 4-acetylaminoantipyrine (4-AcAA)—LGC Standard, UK; ibuprofen 13C3 (IBU-13C3)—Cambridge Isotopes Laboratories, USA; fircoxib-d6 (FIRO-d6) and tolfenamic acid-13C6 (TOL-13C6)—Witega, Germany.

### 3.2. Standard Solutions, Buffers, and Samples

Stock solutions were prepared in MeOH in a concentration of 1 mg/mL and stored at −18 °C (stable for 12 months). Working standard solutions (100 μg/mL) were prepared by diluting the proper stock solution with MeOH and were stable for 6 months. Three mixed standard solutions were prepared by the dilution of suitable aliquots of working standard solutions. The first one was fitted for analyses of muscle tissue; the second was used for milk, and the third one was the internal standard mixed solution used in muscle and milk analyses. The concentration of the internal standard was set to 2 µg/mL for all the compounds used. Acetate buffer used for the enzymatic hydrolysis in the muscle analysis contained sodium acetate (0.33 M) and ascorbic acid (0.01 M), dissolved in ultrapure water. The pH was adjusted to 4.5 with acetic acid.

### 3.3. Instrumentation

During sample preparation, a vortex mixer (Heidolph, Schwabach, Germany), laboratory centrifuge (Sigma 6k15, Darmstadt, Germany), mini-centrifuge (Sigma1-14, Germany), nitrogen evaporator (EVA-EC2-L, VLM, Bielefeld, Germany), and laboratory incubator (e2, Advantage Lab, Darmstadt, Germany) were used. The analysis was performed using a liquid chromatograph (Nexera X2, Shimadzu, Kyoto, Japan) connected to a tandem mass spectrometer (QTrap 5500, Sciex, Concord, ON, Canada), controlled by Analyst 1.7 software.

### 3.4. Optimisation of LC-MS/MS Conditions

For each compound, the mass spectrometry parameters were optimised by injecting working solutions of 10 µg/mL directly into the instrument by an in-built syringe pump. The fragmentation parameters were investigated for each compound individually by monitoring precursor and at least two product ions. For internal standards, only one product ion was monitored. Crucial MS parameters for acquiring and identifying the analysed compounds such as transitions, collision energy, and internal standards for the individual compounds are listed in Table S1 in Supplementary Materials.

### 3.5. Sample Preparation

#### 3.5.1. Milk

10 ± 0.01 g of milk was weighed into a 50 mL-polypropylene centrifuge tube, and 20 μL of IS solution was added. The sample was vortex-mixed and incubated at room temperature for 10 min. Then, 10 mL of ACN was added. The sample was vigorously mixed on the vortex for 1 min. Then, 2 g of ammonium acetate was added. The sample was mixed for 1 min once again and centrifuged for 10 min at −20 °C (4500 rpm). The upper layer of the extract was collected, transferred into a 15 mL-polypropylene tube with 1 g of C18 bulk sorbent, and vortexed once again for 1 min. The sample was centrifuged for 3 min at room temperature (4500 rpm). A volume of 6 mL of the upper layer was collected, evaporated to dryness, and reconstituted in 0.2 mL of DMSO. The sample was transferred into the 1.5 mL Eppendorf tube, centrifuged for 10 min at 14,500 rpm, and put into the autosampler vial and analysed by LC-MS/MS.

#### 3.5.2. Muscle Tissue

Homogenised muscle sample (2 ± 0.01 g) was weighed into a 50 mL polypropylene centrifuge tube, and 20 μL of IS solution was added. The sample was vortex-mixed and left to rest at room temperature for 10 min. Next, 4 mL of acetate buffer (pH = 4.5) and 50 μL of β-glucuronidase were added. The sample was well vortex-mixed for 1 min and left for incubation at 37 °C for 60 min. Next, 10 mL of acetonitrile was added. The sample was vigorously vortex-mixed for 1 min and centrifuged (10 min, 4500 rpm, −20 °C). The upper layer of the extract was collected, transferred into a 15 mL-polypropylene tube with 1 g of C18 bulk sorbent, and vortexed once again for 1 min. The sample was centrifuged for 3 min at 4500 rpm at room temperature. A volume of 6 mL of the upper layer was collected, evaporated to dryness, and reconstituted in 0.2 mL of DMSO. The sample was transferred into the 1.5 mL Eppendorf tube, centrifuged for 10 min at 14,500 rpm, and put into the autosampler vial and analysed by LC-MS/MS.

### 3.6. LC-MS/MS Analysis

The chromatographic separation was carried out on a Luna C8 column (3 μm, 2.1 × 150 mm, Phenomenex, Torrance, CA, USA) connected to a C8 guard column (2.0 × 4 mm, Phenomenex, USA). The gradient was applied with MeOH/ACN (8 + 2, v + v) and 0.01 M ammonium formate, pH = 5.0 (phase B). The initial conditions of the gradient were kept for 6 min at 10% of phase A and 90% of phase B. The phase A content was increased from the 7th minute up to 60% and maintained so until the 11th minute. Next, phase A content was decreased to 10% for re-equilibration. The total run time of the method was 23 min. The flow rate was 0.2 mL min^−1^, the injection volume was 10 μL, and the column temperature was 40 °C.

The optimised multi-residue MS/MS method was based on positive and negative electrospray ionisation (ESI) with nitrogen collision gas (Table 2). The capillary temperature was set to 275 °C, vaporiser temperature was 450 °C. Sheath gas pressure was set to 45 units, and the auxiliary valve flow was set to 30.

### 3.7. Validation Concept

According to CD 2002/657/EC [27], the validation of the method was performed separately for muscle and milk. The parameters considered in this study were selectivity, recovery, precision as a within laboratory reproducibility, the decision limit (CCα), and the detection capability (CCβ). Because the maximum residue level (MRL) varies for different analytes and species, the designed validation protocol was modified to correspond to MRLs regulated by law, recommended concentration, and analytical experience. Thus, the authors decided to set a validation level for analytes without MRL by following the as low as reasonably achievable (ALARA) approach (Table 2 and Table 3). Validation of the method was performed on the National Residue Control Plan samples, which were analysed before validation and identified as blank samples.

#### 3.7.1. Selectivity

To investigate the potential interference of the compounds with the matrix (selectivity), 20 blank muscle samples from different species (horse, bovine, swine) and 20 cow milk samples were analysed. In addition, standard mixtures of other veterinary drugs (antibiotics and anthelmintic) were injected.

#### 3.7.2. Recovery and Precision (Repeatability and Within-Laboratory Reproducibility)

Blank samples of muscle and milk fortified on 0.5, 1.0, and 1.5 of the MRL/validation level were analysed in six replicates for each level. The whole procedure was repeated two more times on two different days. The recovery for selected validation levels and set MRLs should meet the requirements from CD 2002/657/EC [27] for quantitative methods: for mass fraction ≤ 1 µg/kg should be between −50% and +20%, for fraction >1 to 10 µg/kg—between −30% and +10%, and for fraction ≥ 10 µg/kg—between −20% and +10%.

#### 3.7.3. The Decision Limit (CCα) and Detection Capability (CCβ)

These parameters were established by analysing samples from a reproducibility study, fortified at 1.0 mL according to the procedure described in the CD 2002/657/EC [27] and the guidance paper CRLs view on the state of the art analytical methods for National Residue Control Plans [17]. CCα is equal to the concentration of 1.0 mL plus 1.64 times the calculated standard deviation, while CCβ is equal to the CCα plus 1.64 times the standard deviation.

#### 3.7.4. Working Range

The working range was established by preparing three series of matrix-matched calibration curves at the following levels: 0.25, 0.5, 1.0, 2.0, 5.0 MRL/validation level.

## 4. Conclusions

The method described in this paper was successfully developed and validated for analysing residues of NSAIDs in milk and muscle samples. Thanks to sample preparation, LC and MS parameters for both milk and muscle were unified. This method can be easily applied in routine NSAIDs analysis.

## Figures and Tables

**Table 1:** MRL levels established by EU in Commission Regulation No 37/2010 (DC, FIRO, FLU, 5-OH FLU, TOL, MEL, 4-MAA, CPF) [6] and levels * recommended by CRL guidance paper (PBZ, OPB, IBU, NAP, MEF) [17].

Analyte	Animal Species	MRL/Level *	Matrix
Diclofenac (DC)	Bovine	5 μg/kg	Muscle
		0.1 μg/kg	Milk
	Porcine	5 μg/kg	Muscle
Firocoxib (FIRO)	Equidae	10 μg/kg	Muscle
Flunixin (FLU)	Bovine	20 μg/kg	Muscle
	Porcine	50 μg/kg	Muscle
	Equidae	10 μg/kg	Muscle
5-Hydroxyflunixin (5-OH FLU)	Bovine	40 μg/kg	Milk
Tolfenamic acid (TOL)	Bovine, porcine	50 μg/kg	Muscle
	Bovine	50 μg/kg	Milk
Meloxicam (MEL)	Bovine, caprine, porcine, rabbit, Equidae	20 μg/kg	Muscle
	Bovine, caprine	15 μg/kg	Milk
Metamizole (as 4-Methylaminoantipyrin) (4-MAA)	Bovine, porcine, Equidae	100 μg/kg	Muscle
	Bovine	50 μg/kg	Milk
Carprofen (Sum of carprofen and carprofen glucuronide conjugate) (CPF)	Bovine, Equidae	500 μg/kg	Muscle
Phenylbutazone (PBZ) Oxyphenbutazone (OPB)	-	5 μg/kg *	Muscle, milk
Ibuprofen (IBU) Naproxen (NAP) Mefenamic acid (MEF)	-	10 μg/kg *	Muscle, milk

**Table 2:** Validation data for analyses of milk samples.

Analyte	Range (µg/kg)	Validation Level (µg/kg)	Recovery (%)	Precision (CV, %)	ccα (µg/kg)	ccβ (µg/kg)
CELE	1.25–25.0	5.00	105	12.7	3.08	3.86
CPF	1.25–25.0	5.00	104	8.83	2.64	3.09
DC	0.025–0.50	0.10	99.7	16.2	0.15	0.22
FIRO	1.25–25.0	5.00	103	14.6	3.58	4.99
FLU	1.25–25.0	5.00	88.0	5.68	3.21	3.69
FLUF	1.25–25.0	5.00	108	9.54	2.70	3.25
IBU	1.25–25.0	5.00	95.3	11.2	3.61	4.48
KTP	1.25–25.0	5.00	108	6.21	2.66	2.95
MEF	1.25–25.0	5.00	108	11.7	2.67	3.11
MEL	3.75–75.0	15.0	102	8.22	16.8	18.8
NAP	1.25–25.0	5.00	103	10.3	2.95	3.41
NIF	1.25–25.0	5.00	105	10.9	2.55	2.92
OPZ	1.25–25.0	5.00	106	7.72	2.89	3.31
PBZ	1.25–25.0	5.00	106	17.0	3.26	4.32
ROFE	1.25–25.0	5.00	94.5	15.6	3.74	5.03
TOLF	12.5–250	50.0	95.3	13.0	56.0	70.3
4-AA	1.25–25.0	5.00	105	10.9	3.03	3.96
4-AcAA	1.25–25.0	5.00	101	5.51	2.87	3.21
4-FAA	1.25–25.0	5.00	108	12.9	2.76	3.41
4-MAA	12.5–250	50.0	95.3	13.0	56.0	70.3
5-OH FLU	10.0–200	40.0	86.3	13.3	45.9	59.4

**Table 3:** Validation data for analyses of muscle samples.

Analyte	Range (µg/kg)	Validation Level (µg/kg)	Recovery (%)	Precision (CV. %)	ccα (µg/kg)	ccβ (µg/kg)
CELE	1.25–25.0	5.00	93.8	7.14	5.65	6.46
CPF	5.00–1000	20.0	101	13.7	6.45	790
DC	1.25–25.0	5.00	102	6.71	5.59	6.28
FIRO	2.50–50.0	10.0	85.0	7.62	10.4	12.1
FLU	2.50–100	10.0	96.0	16.1	14.2	19.1
FLUF	1.25–25.0	5.00	101	4.73	5.69	7.22
IBU	2.50–50.0	10.0	85.7	13.1	11.0	13.6
KTP	1.25–25.0	5.00	98.6	9.75	5.67	6.45
MEF	5.00–100	20.0	105	6.77	23.6	26.9
MEL	5.00–100	20.0	99.3	7.91	22.6	24.8
NAP	2.50–50.0	10.0	97.7	15.7	12.9	17.7
NIF	1.25–25.0	5.00	104	7.67	5.56	4.48
OPB	1.25–25.0	5.00	103	8.14	5.85	6.96
PBZ	1.25–25.0	5.00	104	12.3	5.88	7.15
ROFE	1.25–25.0	5.00	109	9.63	5.88	7.08
TOLF	12.5–250	50.0	101	6.02	57.1	65.6
4-AA	2.50–50.0	10.0	92.2	15.6	12.8	18.0
4-AcAA	2.50–50.0	10.0	94.6	11.7	12.2	15.8
4-FAA	2.50–50.0	10.0	101	16.1	14.2	19.1
4-MAA	2.50–200	10.0	105	16.6	12.3	146

## Data Availability

The data presented in this study are available on request from the corresponding author.

## Supplementary Materials

The following are available online, Table S1: Mass spectrometry parameters for 21 NSAIDs and corresponding internal standards on QTrap 5500.

## Sample Availability

Samples of the compounds are available from the authors.
