# Supplementary material for: LC-MS/MS Determination of 21 Non-Steroidal Anti-Inflammatory Drugs Residues in Animal Milk and Muscles
Source: Molecules. 2021 Sep 28;26(19):5892. doi: 10.3390/molecules26195892 (PMC8512709; doi:10.3390/molecules26195892)
Supplement: Supplementary file 1 [file molecules-26-05892-s001.zip › molecules-1385097-S1.pdf]

Table S1. Mass spectrometry parameters for 21 NSAIDs and corresponding internal standards on QTrap 5500.

Analyte	Precursor ion (m/z)	Declustering potential (eV)	Product ions (m/z)	Collision energy (eV)	Internal standard
ESI(+)					
DC	296	40	250<br>215	18<br>26	DC-13C6
FIRO	337	143	283<br>237	12<br>23	FIRO-d6
TOL	262	27	209<br>180	38<br>52	TOL-d4
ROFE	315	71	189<br>215	26<br>70	DC-13C6
4-MAA	218	170	56<br>97	44<br>17	FIRO-d6
4-FAA	232	191	83<br>104	27<br>29	FIRO-d6
4-AA	204	185	56<br>83	42<br>19	FIRO-d6
4-AcAA	246	192	104<br>56	42<br>31	FIRO-d6
DC-13C6	302	40	256	18	-
FIRO-d6	343	62	289	13	-
TOL-13C6	268	50	215	37	-
ESI(-)					
CELE	380	-200	316<br>296	-33<br>-38	DIK-13C6
PBZ	307	-70	279<br>131	-26<br>-28	PBZ-13C12
FLU	296	-51	252<br>192	-25<br>-41	FLU-d3
5-OH FLU (milk only)	312	-51	268<br>228	-10<br>-33	FLU-d3
IBU	205	-40	159<br>161	-11<br>-9	IBU-13C3
CPF	272	-40	226<br>228	-37<br>-17	FLU-d3
KTP	253	-15	209<br>197	-10<br>-10	FLU-d3
NIF	281	-20	177<br>216	-42<br>-40	MEL-d3
FLUF	280	-24	236<br>216	-24<br>-24	MEL-d3

MEF	240	-187	196<br>192	-37<br>-22	FLU-d3
MEL	350	-55	286<br>146	-17<br>-25	MEL-d3
NAP	229	-60	185<br>169	-41<br>-11	FLU-d3
OPB	323	-56	295<br>134	-25<br>-35	PBZ-13C12
PBZ-13C12	319	-70	291	-15	-
FLU-d3	298	-125	254	-25	-
IBU-13C3	208	-40	163	-11	-
MEL-d3	353	-50	289	-20	-
